# Global burden of disease in endemic fighting agents, results of an occupational cohort, in a state in northeastern Brazil

**DOI:** 10.47626/1679-4435-2025-1385

**Published:** 2025-07-08

**Authors:** Maria Luiza Almeida Bastos, Marcelo José Monteiro Ferreira

**Affiliations:** 1 Faculdade de Medicina, Universidade Federal do Ceará, Fortaleza, CE, Brazil

**Keywords:** global burden of disease, pesticides, community health workers, occupational medicine., carga global da doença, agentes comunitários de saúde, inseticidas, saúde ocupacional.

## Abstract

**Introduction:**

The health effects of insecticide exposure have been extensively investigated in farmers, but research among endemic fighting agents, as well as health outcomes that may be related to occupational exposure, is still scarce.

**Objectives:**

To understand the global burden of disease in endemic fighting agents throughout their work trajectory and analyze any changes in their morbidity and mortality profile.

**Methods:**

In this historical, census, non-concurrent cohort, data were collected from medical records to measure global burden of disease indicators.

**Results:**

Changes in morbidity patterns were observed over the decades worked. Although traumatic and osteoarticular injuries predominated initially, they were replaced by chronic non-communicable diseases. There was a high burden of mental disorders during the third decade, and neoplasms were an important outcome in the final decade.

**Conclusions:**

These findings are relevant to workers’ health, as they demonstrate a change in disease burden throughout the work trajectory. They also allowed for a critical analysis of the relationship between health conditions and occupational exposures.

## Abstract (pt)

**Introdução:**

Os efeitos à saúde relacionados às exposições aos inseticidas têm sido bastante investigados em agricultores, mas ainda são escassas as pesquisas entre os agentes de combate às endemias, bem como os desfechos em saúde possivelmente relacionados às exposições ocupacionais.

**Objetivos:**

Conhecer a carga global de doenças nos agentes de combate às endemias ao longo de sua trajetória de trabalho e analisar as mudanças no perfil de morbimortalidade.

**Métodos:**

Trata-se de uma coorte histórica, censitária, não concorrente, cujas informações foram colhidas nos prontuários médicos, para mensurar os indicadores de carga global de doenças.

**Resultados:**

Observaram-se mudanças no padrão de morbidade ao longo das décadas laboradas. Inicialmente, com predomínio de lesões traumáticas e osteoarticulares, que foram substituídas por doenças crônicas não transmissíveis. Os transtornos mentais tiveram grande carga no curso da 3ª década, e as neoplasias foram desfechos importantes durante a última década analisada.

**Conclusões:**

Esses achados são relevantes para a saúde do trabalhador, por demonstrarem a mudança na carga de doenças ao longo da trajetória de trabalho. Ainda se permitiu uma análise crítica quanto às condições de saúde apresentadas e sua relação com as exposições ocupacionais.

## INTRODUCTION

Endemic fighting agents (EFAs) are primary care professionals whose scope of activities includes inspecting land, homes, water tanks, gutters, and other possible vector foci of public health importance. Their duties also include the preparation and application of insecticides widely used for vector control in Brazil,^1^ which increases their occupational exposure to these chemical agents.

Historically, the use of insecticides began in Brazil during campaigns against *Aedes aegypti,* initially organochlorines,^2^ especially dichlorodiphenyltrichloroethane (DDT). Over the years, organochlorines have proven extremely harmful to human and environmental health, both as carcinogens^3^ and in association with cognitive impairment in exposed populations.^4^ After they were banned, other classes of insecticides were used in vector control initiatives. However, vector control has involved several other active ingredients over the years: organophosphates, carbamates, pyrethroids, juvenile hormone analogues, chitin inhibitors, and neonicotinoids or mixtures of these agents. Until 2019, organophosphates (e.g. malathion) were prioritized in spatial spraying during campaigns against *Aedes aegypti.*^5,6^ However, they have also been associated with multiple diseases, including cancer,^3^ depression, and anxiety.^7^

Although the health effects of insecticide exposure have been extensively investigated in farmers,^7^ research on EFAs, as well as health outcomes that may be related to occupational exposure, is still scarce. In recent years, these workers have complained of illnesses that may be related to chronic exposure to the insecticides^8^ used in public health campaigns. Given the above, it was deemed necessary to investigate the main diseases that affect these workers and determine whether there is a relationship with occupational exposure.

In search of a methodology that would meet expectations regarding the impact of diseases throughout the career of EFAs, we selected disability-adjusted life years (DALY). This indicator simultaneously measures the impact of mortality and morbidity/disability due to disease and is the sum of two other indicators: years of life lost and years lived with disability. This variable is universally applied in global burden of disease (GBD) studies, whose purpose is to systematically analyze population health.^9^

Therefore, this study investigated GBD in EFAs over the course of their careers. We analyzed the changes in their morbidity and mortality profile, discussing the association between the outcomes and occupational factors, especially exposure to the insecticides used in vector control.

## METHODS

The population of this census cohort, which was not concurrent with the present study, consists of EFAs in the state of Ceará currently linked with the Brazilian Ministry of Health. These data are drawn from the former Fundação Nacional de Saúde (National Health Foundation - FUNASA) and Superintendência das Campanhas de Saúde Pública (Superintendence of Public Health Campaigns - SUCAM). From the 1990s onwards, public health campaigns were decentralized, with responsibility for endemic disease control being shifted to state and municipal governments. After decentralization, no new employees were hired on the federal level, and these EFAs could be the last workers to originate from the former FUNASA and SUCAM.

However, these civil servants remained linked to the federal government, with their employment records maintained by the State Ministry of Health, including medical records related to illness and sick leave. According to information from this agency, there are 1,089 EFAs in the state of Ceará.

After a preliminary assessment of their employment and medical records, it was decided to include only men. Women were excluded because they represented less than 2% of the EFA population (n = 11), which could lead to bias and confounding. Duplicate names, as well as workers with no employment or medical records, were also excluded.

### DATA COLLECTION

The medical records of all EFAs in the Ceará Ministry of Health were analyzed to identify diseases recorded in certificates, medical reports and opinions, and death certificates from admission [considered as the initial observation time (T0)], until the study’s endpoint in 2019.

In this cohort, admission to public service occurred between 1979 and 1986. Due to the differing employment dates, we used the Kaplan-Meyer model (person-time observation) since observation begins from the moment the individual is exposed.^10^ The final observation date for this study was set at December 31, 2019, to avoid confounding factors related to the COVID-19 pandemic.

### GLOBAL BURDEN OF DISEASE INDICATORS

The “years of life lost” variable indicates life lost due to premature death. For each premature death, the number of years lost is counted up to the maximum life expectancy standardized in GBD data.^9,11^ In this study, maximum life expectancy was standardized at 80 years.^9^ To measure this indicator, the cause and age of death was examined in each death certificate. The “years lived with disability” variable indicates the years in which an individual lives with a morbidity from a disease that causes some level of disability.^9^ This indicator consists of the number of incident cases of the disease, multiplied by its mean duration and the burden attributed to disability.^11^ Finally, the “DALY” variable is the sum of the indicators above, calculating the years of life lost adjusted for disability.

Based on these concepts, all diseases recorded during the careers of the EFAs were compiled, as were the days absent from work due to each disease. The diagnoses were identified according to ICD-9 and ICD-10 criteria due to the size of the cohort. After this step, in order to standardize the method, the list of ICD codes were mapped to the respective causes.^12^ The weights applied to the causes are available in the Global Burden of Disease Study 2019.^13^

The length of service was then categorized to determine the disease burden ranking by decade: admission (T0) to 1990 (first decade), 1991 to 2000 (second decade), 2001 to 2010 (third decade) and 2011 to 2019 (fourth decade).

**Figure 1:**
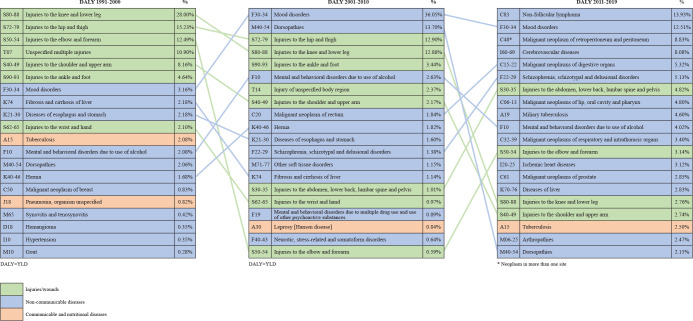
Global burden of disease in endemic fighting agents over the decades worked in Ceará, Brazil, from 1991 to 2019.

This information was entered into a spreadsheet in which each row corresponded to an individual EFA, and the columns were entered according to the variables of interest for calculating DALY. Data analysis and description were performed in Stata 10. Figure 1, constructed using Microsoft Excel, shows the GBD ranking among the EFAs.

### ETHICAL ASPECTS

This is a substudy from a broader project called “Carga de Doenças em Agentes de Combate as Endemias no Estado do Ceará” (Disease Burden Among Endemic Fighting Agents in the State of Ceará), which was approved by the Universidade Federal do Ceará human research ethics committee (decision 3,507,736, August 14, 2019).

## RESULTS

After applying the exclusion criteria, 1,053 EFAs remained, a loss of 3.3%. The results for age and length of service are presented as median and interquartile ranges (IQR) since they were non-normally distributed. The median age at admission was 24.7 years (IQR = 4.14; 18-39). At the study’s endpoint, the population’s median age was 59.7 years (IQR = 4.66; 46-76) and the median length of service was 35 years (IQR = 3.0; 32-44).

The cohort’s disease burden over the decades is presented in Figure 1. During the first decade of work, no diseases were associated with work absences, although from the second decade onwards, absences due to fractures, sprains, and acute osteoarticular injuries began to occur (Figure 1). During the third decade, mood disorders were the leading cause of DALY, higher than either back injuries or acute osteoarticular injuries. In the fourth decade, neoplasms accounted for a significant portion of DALY, especially non-Hodgkin’s lymphoma, retroperitoneal neoplasms, neoplasms of digestive organs, and malignant neoplasms of the lip, oral cavity, and oropharynx.

Eight deaths were recorded during the study period: in four the cause was undefined, two were due to respiratory system neoplasms, one was due to cirrhosis, and another was due to acute myocardial infarction. These results are described in Figure 1, including the GBD ranking and the DALY trend.

## DISCUSSION

This study identified disease profile changes during the career of Brazilian EFAs, which, surprisingly, included a high burden from mood disorders and disorders related to alcohol use, in addition to a relevant burden from neoplasms during the final decade of work.

This study is innovative in that it is an occupational cohort, albeit retrospective, including EFAs from the former SUCAM agency. Although these professionals played an important role in the control of vector-borne diseases, they faced many occupational health adversities, including exposure to various insecticides/pesticides used over the years in public health campaigns.^8^

It should be noted that using the GBD methodology to assess the longitudinal impact of disease is also a cutting-edge model in the current literature. Studies like this are still scarce in working populations, despite their great analytical potential. The results of this study show a change in the morbidity profile of EFAs. When younger, the most relevant diseases were musculoskeletal, which cause a high burden worldwide.^14^ In Brazil, they are among the main causes of work disability. It is worth noting that the scope of CA activities includes home visits, inspecting water tanks and, often, long working hours,^5^ which may corroborate the prevalence of these conditions. Another Brazilian study demonstrated that musculoskeletal complaints are common among EFAs.^15^

Mood and mental disorders due to alcohol use also accounted for a significant share of DALYs between 1991 and 2000, but their increase became evident in the following decade. Mood disorders rose from seventh to first place in the ranking, and mental disorders due to alcohol use increased from twelfth to sixth place.

Mental disorders require complex analyses, but the role of work as a social determinant in the disease process is indisputable. The occupational situation of EFAs deserves attention due to the high work demand during vector control campaigns, issues related to low professional recognition^15^ and exposure to insecticides.^1-3,6,15^ Qualitative research has demonstrated these professionals’ lack of perceived recognition: “We are the garbage dump,” they reported.^15^

Some studies have already associated insecticide exposure with the emergence of neurodegenerative and mental disorders.^16,17^ National research on this population has also highlighted the high burden of mood disorders, stress-related disorders, and alcohol abuse, which directly affect years lived with disability.^18^ Furthermore, the prevalence of alcoholism among EFAs has also been documented, which may be related to the workers’ distance from their family during campaigns or even a feeling of lack of prestige at work.^1,19^ There is also evidence of an increased risk of alcohol abuse in workers exposed to pesticides.^20^

As previously mentioned, we found a significant change in morbidity and mortality patterns over the career of EFAs, and we highlight the high burden of neoplasms in the final decade (2011 to 2019). These results corroborate estimates from GBD 2019, which warn of millions of cases of cancer, including: lung, colon, rectal, prostate, stomach, and non-Hodgkin lymphoma. National surveys have shown high mortality rates from cancer, including breast, prostate, and head and neck tumors in agricultural regions.^21^ Importantly, a study conducted in Ceará reported a high rate of hospitalizations and mortality due to neoplasms in regions with intensive pesticide use.^22^

The high rate of neoplasms related to pesticide exposure is consistent with the results of the present study. Regarding non-Hodgkin’s lymphoma, it has been reported in the national and international literature that exposure to organochlorines^23^ and organophosphates^24,25^ is an important risk factor. Regulatory decrees have established occupational links to exposure.^25,26^

Regarding digestive system cancer, although the literature points to possible associations, the findings are still inconclusive.^25^ Head and neck tumors such as lip and oral cavity cancer are generally squamous cell cancers, whose major risk factor is smoking. However, unprotected sun exposure also has a major impact on the incidence of these diseases.^26^ It is important to remember that EFAs carry out field activities, covering large open areas exposed to the sun.^5^

Despite being the most common neoplasm in the male population, there was a high incidence of prostate cancer among the EFAs. This cancer ranked fourteenth in the GBD ranking. Longitudinal studies have shown a higher risk of prostate cancer among individuals with a history of pesticide exposure,^27^ particularly malathion.^28,29^ In Brazil, it has also been recognized that insecticides are risk factors for prostate cancer.^28,30^

Oncological research has attributed more than 30% of neoplasms to modifiable factors.^30^ However, it is still believed that the magnitude of work-related cancer is underestimated, perhaps due to the lack of information on exposure or low reporting of occupational cancer.^3^ Therefore, occupational and environmental contexts have a considerable weight in preventing these diseases, and treating them reductionistically makes it extremely difficult to apply measures capable of reducing their incidence.

Finally, we believe that the results of this study can broaden perspectives on occupational disease among EFAs. GBD studies can help guide health initiatives, including those on occupational health and social security, to minimize the social and economic burden of diseases with high morbidity and mortality.

### STUDY LIMITATIONS

The low quality of the data on work processes, occupational exposure, personal protective equipment use, lifestyle habits, and family history in the medical records prevented analysis of many explanatory variables. A questionnaire was developed to obtain such information, but adherence was low among workers. Many reported memory bias, in addition to not wanting to provide information about education, socioeconomic, or occupational conditions. However, the data collected from the medical records contribute to the internal validity of the research. This method could also be reproduced in other occupational health studies, thus contributing to its external validity.

## CONCLUSIONS

This study identified the previously unknown disease burden among EFAs, including a transition in causes over their careers. These results could contribute to a new perspective on worker health, especially occupational exposure in public health campaigns. The observation that neoplasms emerged during the fourth decade of work was only possible by applying epidemiological concepts in a longitudinal study, given the great length of time elapsed between exposure and outcome. Our results for both neoplastic and neuropsychiatric outcomes were consistent with other studies, which raises the question of a relationship between EFA work processes and occupational exposure to insecticides in vector control campaigns.

Research such as this has enormous potential for planning and monitoring occupational health initiatives, especially for EFAs. Our methodology also allows the assessment of interventions and changes in morbidity over the follow-up period, which can translate into more assertive strategies for economically active populations.

### CLARIFICATIONS

For the purposes of this study insecticides were considered a class of pesticides used to control both arthropods and insects.
